# Increased serum carboxylesterase-1 levels are associated with metabolic dysfunction associated steatotic liver disease and metabolic syndrome in children with obesity

**DOI:** 10.1186/s13052-024-01733-7

**Published:** 2024-09-04

**Authors:** Huanyu Wang, Shimin Wu, Ying Weng, Xi Yang, Ling Hou, Yan Liang, Wei Wu, Yanqin Ying, Feng Ye, Xiaoping Luo

**Affiliations:** 1 Department of Pediatrics, Tongji Hospital, Tongji Medical College, Huazhong University of Science and Technology, Wuhan, China; 2 Hubei Key Laboratory of Pediatric Genetic Metabolic and Endocrine Rare Diseases, Wuhan, China

**Keywords:** Children, Obesity, Carboxylesterase-1, Metabolic syndrome, Metabolic dysfunction associated steatotic liver disease, Biomarker

## Abstract

**Background:**

Carboxylesterase 1(CES1) is expressed mainly in the liver and adipose tissue and is highly hypothesized to play an essential role in metabolism. Our study aimed to investigate the association between CES1 and metabolic syndrome (MetS) and metabolic dysfunction associated steatotic liver disease (MASLD) in children with obesity in China.

**Methods:**

This study included 72 children with obesity aged 6-13years (including 25(35%) diagnosed as MetS and 36(50%) diagnosed as MASLD). All subjects were measured in anthropometry, serum level of biochemical parameters related to obesity, circumstance levels of insulin-like growth factor1, adipokines (adiponectin, leptin and growth differentiation factor 15) and CES1.

**Results:**

Higher serum CES1 level were found in the MetS group (*P* = 0.004) and the MASLD group (*P* < 0.001) of children with obesity. Serum CES1 levels were positively correlated with alanine aminotransferase, aspartate aminotransferase, triglyceride, cholesterol, low-density lipoprotein cholesterol, GDF15, Leptin and negatively correlated with high-density lipoprotein cholesterol, adiponectin and IGF1. We also found a multivariable logistic regression analysis of MASLD and MetS predicted by CES1 significantly (MASLD *P* < 0.01, MetS *P* < 0.05). The combination of CES1, sex, age and BMI *Z-*score showed a sensitivity and specificity of 92.7% for the identification of MASLD and 78.6% for the identification of MetS. The cutoff for CES1 of MASLD is 56.30 ng/mL and of MetS is 97.79 ng/mL.

**Conclusions:**

CES1 is associated with an increasing risk of MetS and MASLD and can be established as a biomarker for metabolic syndrome and MASLD of children with obesity.

**Supplementary Information:**

The online version contains supplementary material available at 10.1186/s13052-024-01733-7.

## Backgound

Worldwide, the prevalence of childhood obesity is increasing year by year [1]. Childhood obesity is associated with many metabolic disorders, including metabolic dysfunction associated steatotic liver disease (MASLD) and metabolic syndrome (MetS) [2]. MASLD is defined as excess lipid infiltration of the hepatic parenchymal cells with one cardiometabolic risk factor (CMRF) and without other pathogenic factors [3, 4]. MetS is a group of clinical symptoms characterized by abdominal obesity, hyperglycemia, hypertension and dyslipidemia [5]. Both MASLD and MetS can have adverse consequences without early intervention, so it is necessary for children with obesity to identify risk factors as early as possible [6]. However, the exact diagnostic criteria for MetS in childhood and the exact therapeutic indications and strategies for pediatric MASLD are not variable. Thus, it is extremely important to define new biomarkers for the diagnosis of MetS and MASLD in children with obesity.

Carboxylesterase 1 (CES1) is a crucial serine hydrolase located mainly in parenchymal hepatic cells and adipocytes [7, 8]. CES1 works primarily on hydrolyzing endogenous lipids, including cholesterol esters, triglycerides, and promotes lipid storage [9]. Previous studies have demonstrated that CES1 gene expressions increased in adipose tissues of obese animals and during differentiation of adipocytes [8, 10]. CES1 could also be found in the medium supernatant of human brown adipocytes and increased after norepinephrine stimulation [11]. CES1 also participated in obesity-induced hepatic steatosis, inactivation, and CES1 deficiency in obesity animal models induced by high-fat diet could improve the development of liver steatosis and steatohepatitis [12]. Numerous studies in human adults have demonstrated that increased gene expression of CES1could be observed in MASLD, NASH patients and correlated with obesity and related cardiovascular risk factors [13]. However, studies focus on CES1 levels in the serum of objects with obesity are rare and it is unknown how CES1 levels changed in children with obesity.

In this study, we focus on investigating the association between serum CES1 level and MASLD, MetS, in children and adolescents in China with obesity. We found that levels of CES1 increased in both the MASLD and MetS group. Furthermore, we identified that the serum CES1 level had a diagnosis value for MASLD and MetS in children with obesity.

## Methods

### Subjects

A total of 72 children (44 boys, 28 girls) with obesity aged 6–13 years were recruited from the Department of Pediatrics at Tongji Hospital from January 2023 to September 2023. Parents of all children signed an informed consent before inclusion, and all procedures of this study were approved by the Tongji Hospital Ethics Committee. The criterion for the diagnosis of obesity in children and adolescents was using criteria of the World Health Organization [14]. The exclusion criteria were: being diagnosed with metabolic disease of heredity, taking drugs that could influence lipid metabolism and glucose metabolism, leading to liver function damage in the last 6 months, having acute infection, and having a history of alcohol drinking.

### Anthropometric assessments

All subjects complemented the anthropometric assessments including height (cm), body weight (kg) and waist circumference (WC) (cm) by experienced physicians using standard procedures [15]. Body mass index (BMI) was calculated by weight (kg) divided by the square of one’s height (cm). BMI *Z*-score: *Z* = [(BMI/M) L − 1]/ (L × S) [the median (M), coefficient of variation (S) and skewness (L)] according to Chinese criteria [16]. The waist -to- height ratio was calculated by dividing waist circumference (cm) by height (cm). Blood pressure was measured after subjects had rested for 5 min and three times for each subject using the same mercury sphygmomanometer.

### Biochemical evaluations and ELISAs

Blood samples were collected after fasting for 12 h and centrifuged at 3000 g for 15 min. Serum samples were stored at -80℃ for further biochemical and protein detection. Serum levels of alanine aminotransferase (ALT), aspartate aminotransferase (AST), total triglyceride (TG), total cholesterol (TC), high-density lipoprotein cholesterol (HDL-C), low-density lipoprotein cholesterol (LDL-C), fasting blood glucose (FBG), fasting blood insulin (FBI) were measured by automatic biochemical analyzer. Homeostasis model assessment of insulin resistance (HOMA-IR) was done using the formula as follows: FBG (mmol/L) × FBI (mU/L)/22.5. Serum levels of adiponectin, leptin, GDF15, IGF-1, CES1 were measured using enzyme-linked immunosorbent assay ELESA kits (Boster Biological, Pleasanton, CA) following the manufacturer’s protocol.

### Recruitment

Hepatic steatosis was checked by abdominal ultrasonography and MASLD was defined by standard criteria [3, 4]. MetS was defined using the criteria proposed by the International Diabetes Institute [17] for children age 10 to 16 years that consisted of central obesity (waist circumference ≥ 90th percentile of children of the same age and sex) and having any two of four risk factors: hyperglycemia, hypertension, low HDL-C and high triglycerides. For children between 6 and 10 years, the cut points for cardiovascular disease (CVD) risk factors were used to replace diagnosis of MetS which was composed of obesity (BMI ≥ 95th percentile or waist circumference ≥ 95th percentile) and having two of three risk factors: hypertension (blood pressure ≥ 95th percentile), dyslipidemia (HDL-C < 1.03 mmol/L, non-HDL-C ≥ 3.76 mmol/L, TG ≥ 1.47 mmol/L), hyperglycemia (FBG ≥ 5.6 mmol/L) [18].

### Statistical analysis

Statistics were performed using SPSS 23 (SPSS, Chicago, Illinois) and a *P* value < 0.05 was considered significant. Shapiro-wilk tests were used to test the normality of the data distribution. Data for variables confirming a normal distribution were presented as mean ± standard deviation and Student’s t test was used for comparisons between two groups. Data for variable with a Skewed distribution were presented as median (interquartile range) and Mann–Whitney U tests were used for comparisons between two groups. Pearson’s correlations were performed to evaluate the association of serum CES1 levels with metabolic risk factor levels, adiponectin, leptin, GDF15, and IGF-1 levels in serum after the data for variables not confirming a normal distribution performed a logarithmic transformation. Partial correlation coefficients were used for data adjusted for sex and age.

Binary logistic regression was performed to identify variables independently associated with serum CES1 levels and confounders were chosen by 3 important criteria [19]. A binary logistic regression model was used to identify the value of serum carboxylesterase 1 levels on recognizing non-alcoholic fatty liver disease and metabolic syndrome. Receiver operating characteristic (ROC) curves were performed to determine the risk of MASLD and MetS according to serum levels of CES1.

## Results

### Clinical characteristics of subjects with obesity

**Fig. 1:**
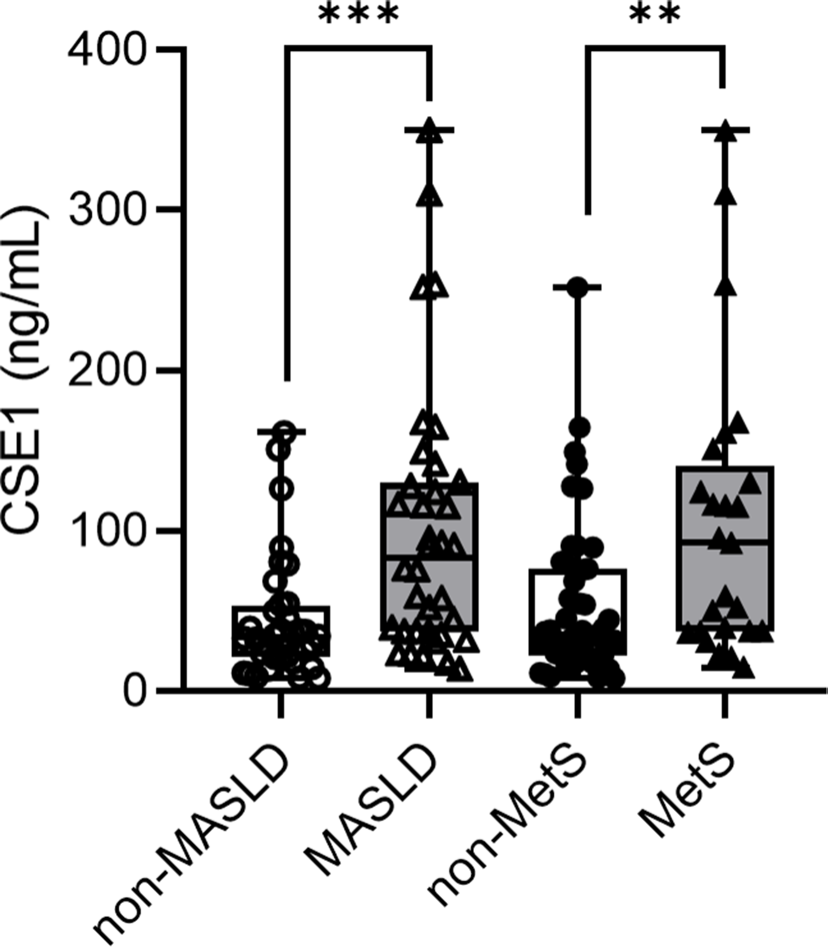
Plasma levels of CES1 comparing non-MASLD to MASLD individuals and non-MetS to MetS individuals. Statistical assessment was considered statistically significant at *P < 0.05, **P < 0.01

A total of 72 children with obesity (44 boys and 28 girls) are included. Among these children, 50% (36/72) had MASLD and 34.7% (25/72) had metabolic syndrome (MetS). The clinical measurement and biochemical detection characteristics of the study objects are presented in Table 1. Boys were more likely to have MASLD (*P =* 0.004) and older children were more likely to have MetS (*P* = 0.036). As expected, the weight, waist circumference, and BMI values were significantly higher in both the MASLD group and the MetS group. Serum levels of ALT, AST, TG, LDL-C, fasting insulin, HOMA-IR were significantly higher and HDL-C were lower in both groups. However, TC levels and BMI *Z*-score were only significantly higher in children with MASLD. The circulating levels of GDF15 was markedly higher in subjects with MASLD (*P* < 0.001), MetS (*P* < 0.001). Interestingly, the serum level of CES1 was significantly higher in the MASLD group (*P* < 0.001) and the MetS group (*P* = 0.004) (Fig. 1). However, serum leptin levels were only significantly higher in patients with MASLD and serum adiponectin levels were lower in both the MASLD and MetS group, but there were no statistic differences.

**Table 1:** Characteristics of obesity subjects according to MASLD and MetS

Variables	All	MASLD			Metabolic syndrome		
		No	Yes	*P*-value	No	Yes	*P*-value
N (boys/girls)	72(44/28)	36(16/20)	36(28/8)	0.004*	47(28/19)	25(16/9)	0.7
Age (years)^2^	11(9,12)	10(9,11)	10(9,11)	0.5	10(9,11)	12(9.5,13)	0.036*
Weight (kg) ^2^	65 (53, 76)	57 (48, 68)	70 (63, 88)	< 0.001*	61 (51, 70)	75 (64, 90)	0.002*
WC (cm) ^2^	89 (83, 97)	84 (77, 88)	96 (89, 108)	< 0.001*	86 (81, 94)	96 (89, 105)	0.003*
WHtR ^1^	0.60 ± 0.7	0.56 ± 0.05	0.65 ± 0.07	< 0.001*	0.59 ± 0.07	0.63 ± 0.08	0.057
SBP (mmHg) ^1^	110.75 ± 13.19	106.67 ± 12.32	114.83 ± 12.91	0.008*	106.27 ± 11.40	119.16 ± 12.35	< 0.001*
DBP (mmHg) ^1^	71.51 ± 9.27	67.94 ± 8.02	75.08 ± 9.15	0.001*	69.26 ± 9.10	75.76 ± 8.16	0.004*
BMI (kg/m^2^) ^1^	28.69 ± 5.28	25.73 ± 4.03	31.66 ± 4.72	< 0.001*	27.48 ± 5.06	31.00 ± 5.00	0.006*
BMI *Z*-score ^1^	3.07 ± 0.72	2.80 ± 0.63	3.34 ± 0.71	0.001*	2.97 ± 0.70	3.25 ± 0.73	1.12
ALT (U/L) ^2^	21 (14, 38)	14 (11, 20)	36 (21, 68)	< 0.001*	18 (12, 28)	31 (20, 107)	< 0.001*
AST (U/L) ^2^	22 (20, 34)	20 (18, 23)	26 (21, 40)	< 0.001*	21 (20, 25)	30 (20, 44)	0.03*
TG (mmol/L) ^2^	1.05 (0.80, 1.54)	0.91 (0.67, 1.19)	1.25 (0.99, 1.64)	0.004*	0.94 (0.69, 1.14)	1.54 (1.18, 1.72)	< 0.001*
TC (mmol/L) ^1^	3.88 ± 0.67	3.70 ± 0.60	4.04 ± 0.71	0.03*	3.79 ± 0.61	4.03 ± 0.76	0.144
HDL-C (mmol/L) ^1^	1.13 ± 0.27	1.22 ± 0.26	1.04 ± 0.24	0.003*	1.20 ± 0.27	1.00 ± 0.18	< 0.001*
LDL-C (mmol/L) ^1^	2.49 ± 0.64	2.24 ± 0.54	2.74 ± 0.64	0.001*	2.36 ± 0.55	2.73 ± 0.73	0.016*
FBG (mmol/L) ^2^	4.86 (4.58, 5.11)	4.80 (4.62, 5.05)	4.89 (4.57, 5.15)	0.7	4.75 (4.60, 5.12)	4.91 (4.58, 5.10)	0.7
FBI (mU/L) ^2^	20 (12, 26)	15 (9, 21)	24 (14, 33)	< 0.001*	14 (10, 22)	26 (17, 38)	< 0.001*
HOMA-IR ^2^	4.46 (2.55, 6.15)	3.50 (1.84, 4.60)	5.30 (2.96, 7.25)	< 0.001*	3.03 (2.12, 4.96)	6.09 (4.71, 7.67)	< 0.001*
Adiponectin (ug/mL) ^2^	11 (7, 14)	12 (7, 16)	9 (7, 13)	0.11	12 (7, 15)	9 (6, 13)	0.089
Leptin (ng/mL) ^2^	24 (16, 35)	18 (11, 28)	28 (20, 45)	0.003*	22 (12, 33)	25 (19, 38)	0.4
GDF15 (ng/mL) ^2^	209 (169, 267)	175 (156, 218)	240 (201, 315)	< 0.001*	190 (161, 226)	278 (204, 338)	< 0.001*
IGF-1 (ng/mL) ^2^	212 (138, 294)	255 (167, 314)	178 (121, 265)	0.016*	222 (148, 298)	195 (124, 290)	0.4
CES1 (ng/mL) ^2^	39 (27, 93)	34 (21, 51)	84 (37, 129)	< 0.001*	35 (23, 76)	93 (37, 130)	0.004*

^1^Data are presented as mean ± standard deviation. Student’s t test was used for the comparison. ^*^P < 0.05

^2^Data are presented as median (interquartile range). Mann–Whitney U tests were used for the comparisons. ^*^P < 0.05

### Increased serum CES1 levels were associated with metabolic risk factors and adiposity in children with obesity

**Fig. 2:**
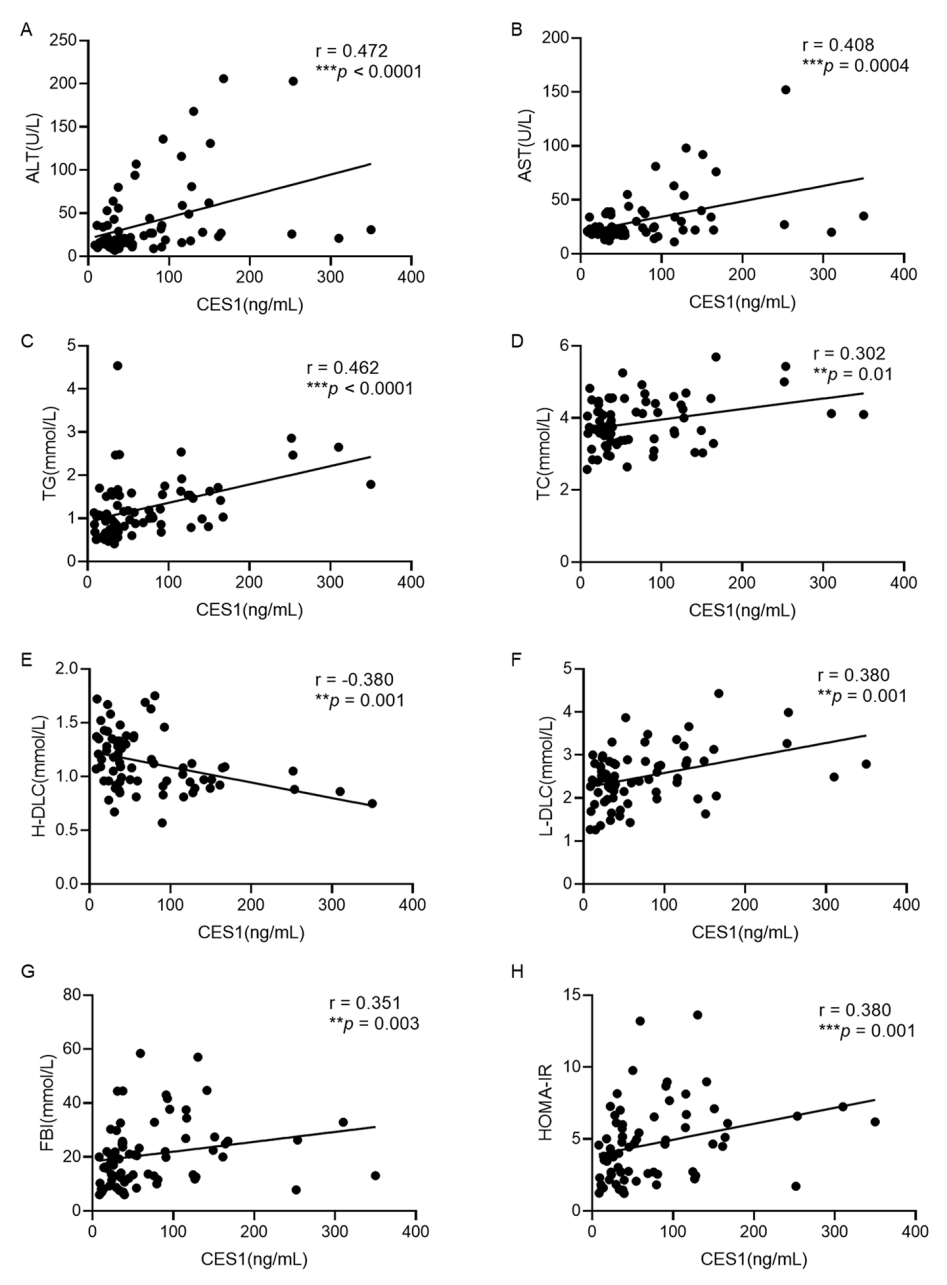
Correlation analysis between CES1 and metabolic risk factors. (**A**) ALT, (**B**) AST, (**C**) TG, (**D**) TC, (**E**) H-DLC, (**F**) L-DLC, (**G**) FBI, (**H**) HOMA-IR. Statistical assessment was 2-sided and considered statistically significant at *P < 0.05, **P < 0.01, ***P < 0.001

**Fig. 3:**
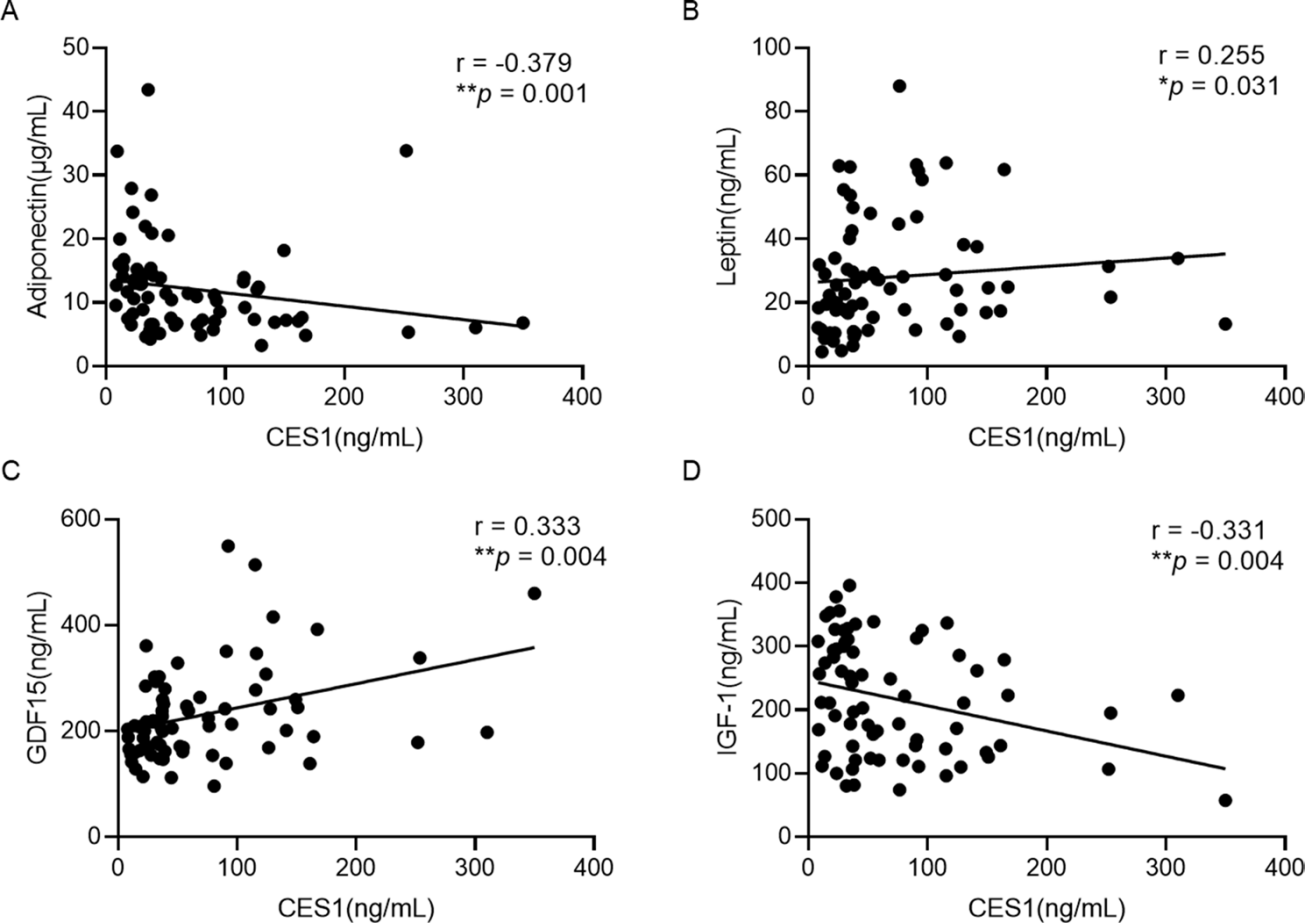
Correlation analysis between CES1 and obesity-related secreted proteins. (**A**) Adiponectin, (**B**) Leptin, (**C**) GDF15, (**D**) IGF-1. Spearman correlation coefficient was used. Statistical assessment was 2-sided and considered statistically significant at *P < 0.05, **P < 0.01

To analyze the relationship between serum CES1 and obesity-related factors, Pearson’s correlations were performed. As shown in Extend Table 1, the age of subjects with obesity was positively associated with serum CES1 levels (*P* = 0.024). In terms of anthropometric indices, such as WHtR, BMI *Z*-score were not associated with serum CES1 levels and weight (*P* = 0.028), WC (*P* = 0.026), BMI (*P* = 0.041) was positively associated with serum CES1 levels (Extend Table 1). Serum CES1 levels were positively correlated with ALT, AST, TG, TC, LDL-C, FBI, HOMA-IR and negatively correlated with HDL-C (Fig. 2). Surprisingly, circumstance CES1 levels were positively associated with leptin, GDF15 and negatively associated with adiponectin, IGF1 (Fig. 3). After adjusted for age and sex, most correlations remained (*P* < 0.05) beside weight, WC, BMI, leptin (*P* > 0.05).

### Increased circumstance CES1 levels were related to increased risk of MASLD and MetS

To conform the relationship between serum CES1 levels and MASLD or MetS, binary logistic regression analyses (*n* = 72; Table 2) were further made to identify the risks of the diseases belong and correctly adjusted by confounders such as sex, age, weight, waist circumference and BMI (Table 2) [19]. The risk of MASLD was increased 1.95 times per 1 SD increase in serum CES1 levels (log transformed) (OR: 2.95; 95% CI, 1.598–5.443, *P* = 0.001). Increased serum CES1 was significantly associated with an increased risk of MetS (OR: 2.255; 95% CI, 1.277–3.981, *P* = 0.005). After adjustment for confounders, higher serum levels of CES1 were still independently related to higher MASLD risk (OR:3.561; 95% CI, 1.598–7.938, *P* = 0.002) and MetS risk (OR: 2.040; 95% CI, 1.103–3.773, *P* = 0.023).

**Table 2:** Effect of serum carboxylesterase 1 levels on metabolic dysfunction–associated steatotic liver disease and metabolic syndrome

	MASLD			Metabolic syndrome		
	β(SE)	OR ( 95%CI )	*P*	β(SE)	OR ( 95%CI )	*P*
Model1^1^	1.082(0.313)	2.950(1.598,5.443)	0.001	0.813(0.290)	2.255(1.277,3.981)	0.005
Model2^2^	1.344(0.379)	3.834(1.826,8.053)	< 0.001	0.707(0.298)	2.028(1.131,3.637)	0.018
Model3^3^	1.270(0.409)	3.561(1.598,7.938)	0.002	0.713(0.314)	2.040(1.103,3.773)	0.023

^1^Model1, unadjusted

^2^Model2, adjusted for age and sex

^3^Model3, adjusted for age, sex, body mass index, waist circumference, weight

### Circulating concentrations of CES1 as a predictor for MASLD and MetS in children with obesity

**Fig. 4:**
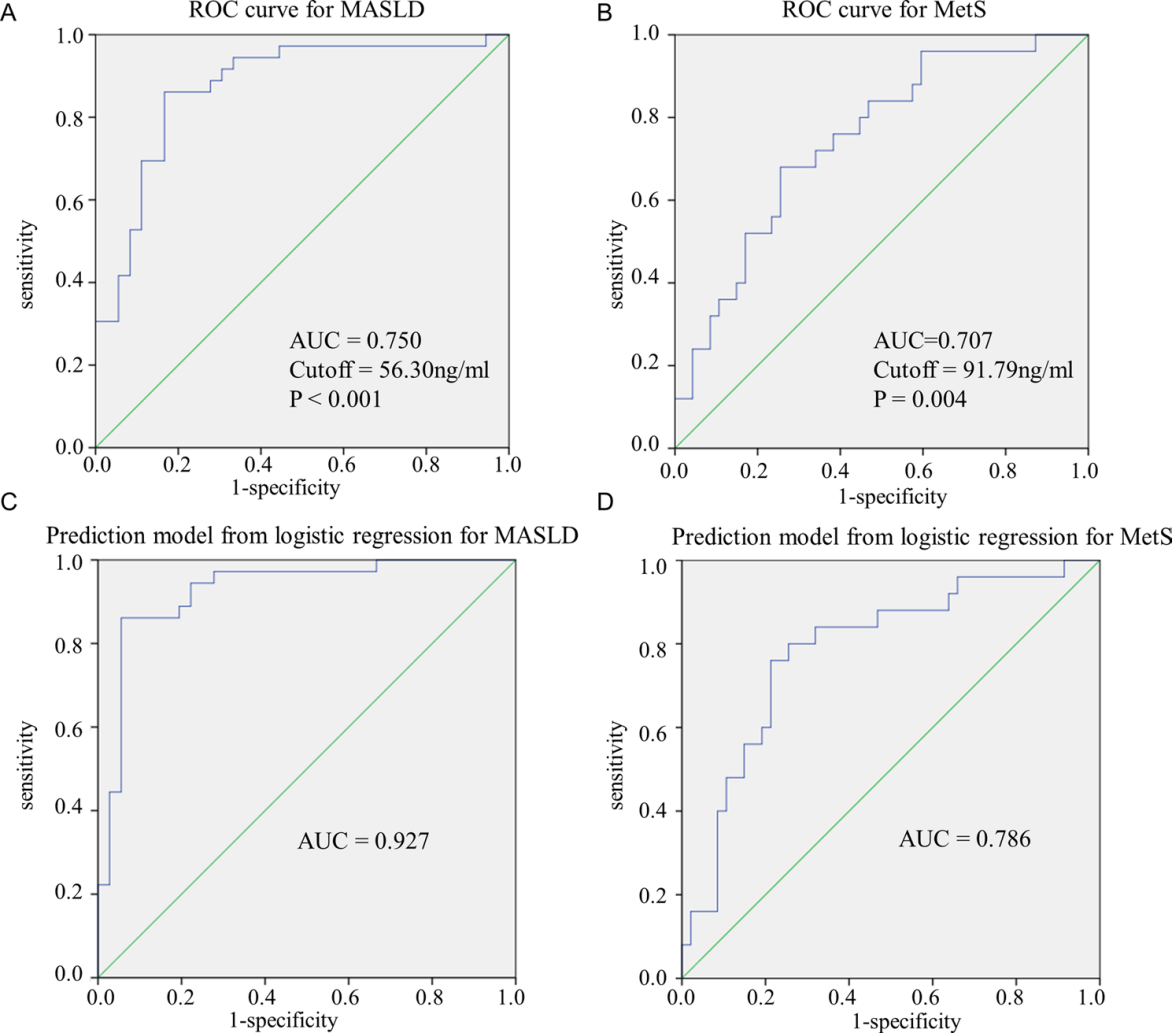
Receiver operating characteristic (ROC) curve for metabolic dysfunction associated steatotic liver disease (MASLD) (**A**) and metabolism syndrome (MetS) (**B**) according to serum CES1 levels. Receiver operating characteristic (ROC) analyses of logistic regression prediction models after combination of CES1, age, sex and BMI *Z*-score for MASLD (**C**) and MetS (**D**)

Following, we used the ROC curve to assess the capacity of serum CES1 levels used to discriminate children with or without MASLD or MetS. The AUC of circulation CES1 levels in above-mentioned children with MASLD shown in Fig. 4 was 75% (ROC-AUC = 0.750, 95% CI, 0.638-0 0.862, *P* < 0.001); the optimal cutoff point of serum CES1 levels for diagnosing MASLD was 56.30 ng/ml (specificity = 0.806, sensitivity = 0.611; Youden’s index = 0.417). The AUC of serum CES1 levels in children diagnosed with MetS was 70.7% (ROC-AUC = 0.707, 95% CI, 0.581–0.833, *P* = 0.004); the optimal cutoff point of serum CES1 levels for diagnosis of MetS was 97.79 ng/ml (specificity = 0.872, sensitivity = 0.520, Youden’s index = 0.392).

After a combination of CES1, BMI *Z*-score, age and sex for the logistic regression prediction model, the percentage of correct predictions for MASLD was 88.9% (ROC-AUC = 0.927, 95% CI, 0.862–0.991, *P* < 0.001) and for MetS was72.6% (ROC-AUC = 0.786, 95% CI, 0.673–0.898, *P* < 0.001).

## Discussion

In this study, we first presented a higher serum level of CES1 in both the MASLD and MetS groups of children with obesity and the serum CES1 levels were significantly associated with lipid metabolic disturbance, abnormal glucose metabolism and circulating adipokines. Furthermore, elevated serum CES1 levels were independently associated with higher risk of MASLD and MetS in children with obesity in China. These findings indicated that CES1 as a new biomarker of metabolic disorder in children with obesity may play a crucial role in the progression of MASLD and MetS.

Obesity is caused by excessive energy intake and large amounts of lipids deposited in fat tissue, leading to hypertrophy of adipocytes and inflammation of adipose tissue [20]. Adipokines are a group of proteins secreted by adipocytes that can be dysregulated during obesity and contribute to obesity-related disorders such as MASLD and MetS [21]. Therefore, the study of specific adipokines in patients with obesity of their secretion levels and mechanism pathways is helpful for the treatment or prevention of the development of metabolic disorders.

CES1 is a carboxylesterase that belongs to the α/β-hydrolase fold family of proteins and is expressed mainly in the liver and also express in adipose tissue. CES1 has hydrolase activity of triglyceride (TG), cholesteryl ester (CE) and also participates in the metabolism of lipid droplets (LDs) which is predicted to play a role in energy homeostasis [22–24]. CES1 expression was increased during the differentiation of adipocytes and is higher in adipose tissue of patients with obesity [8, 25]. Ces1 deficiency in mice leads to a decrease in blood triglyceride levels, an improvement glucose metabolism and increased energy expenditure [26, 27]. Treatment of Ces1 inhibitors to obesity mice induced by a high fat diet and *db/db* mice improved multiple features of metabolic disorders, including decreased weight gain, improved lipid and glucose metabolisms and improved liver steatosis [11]. All of the studies presented above illustrated that an increased level of CES1 may have harmful effect on the development of obesity-related disorders and inhibiting the activity of CES1 may improve metabolic disorders of obesity [28]. In our study, we found that serum CES1 levels were significantly higher in children with obesity diagnosed as MASLD or MetS which was consistent with previous studies [29].

It is well known that obesity can lead to excess lipid accumulation in liver and adipose tissues that is associated with metabolic risk factors such as waist circumstances, BMI, insulin resistance, hyperlipidemia, and abnormal liver function [20, 30]. In this study, we indicated that obesity indices (weight, WC, BMI) and metabolic risk factors (ALT, AST, TG, TC, LDL-C, FBI, HOMA-IR values) were higher in children with obesity with MASLD or MetS than without. Moreover, GDF15 which been positively associated with NAFLD-NASH progression, was increased in children with obesity of MASLD or MetS and leptin increased only in subjects of MASLD but not MetS [31].

The most interesting finding in this study was that serum CES1 levels were significantly associated with lipid metabolism (ALT, AST, TG, TC, LDL-C), glucose metabolism (FBI, HOMA-IR) and obesity-related secreted proteins (adiponectin, leptin, GDF15, IGF-1). As previously reported, CES1 could participate in triglyceride, cholesterol ester hydrolysis, and the lipid drop formation in liver parenchymal cells and adipocytes [9]. Overexpression of Ces1 in mice could increase liver lipolytic activity leading to higher TG level in serum [32]. Insulin resistance played a crucial role in development of MASLD, MetS and other metabolic disorders, and HOMA-IR is the most common method used to assess insulin resistance [33]. In studies in adult individuals, the serum leptin level is positively associated with metabolic indices including increased weight, insulin resistance, and inflammation [34]. Adiponectin is predicted as a possible antidiabetic adipokine which is considered as an important correlative marker of the sensitivity to systemic insulin resistance and the action of adipocytes [35]. GDF15 is a newly secreted adipokine by both liver and adipocytes, and could be a prognostic biomarker to predict fibrosis in NAFLD [36]. IGF-1 was secreted primarily by the liver and was decreased in patients with NAFLD, which could play a protective role in the liver fibrosis process [37]. According to the above, we believed that serum CES1 in children with obesity was mainly secreted from liver and adipose tissues and may play an irreplaceable role in the progression of insulin resistance and NAFLD fibrosis.

To further explore the association between serum CES1 level and the risk of MASLD and MetS in children with obesity, a binary logistic regression model was used to analyze including sex, age, and obesity indices (BMI, weight, WC). The risk of MASLD was increased 1.95 times and the risk of MetS was increased 1.25 times per 1 SD increase in serum CES1 levels. Furthermore, the ROC curve was conducted to assess the capacity of serum CES1 levels used to discriminate children with or without MASLD or MetS. And the cut-off point for CES1 of MASLD is 56.30 ng/ml and of MetS is 97.79 ng/ml.

Although our study found a new biomarker for both MASLD and MetS in children with obesity, it still has few limitations. First, the study used only children with obesity without normal individuals, which was insufficient to assess serum CES1 levels between the obesity group and normal individual groups. Second, it is difficult to diagnose MetS in children earlier than 10 years old, instead, we used CVD risk factors that may remain controversial. Third, we did not distinguish the different stage of MASLD by the limitation of ultrasound diagnosis. More studies are needed to test CES1 level in the development of MASLD.

## Conclusion

In summary, this study indicated that serum CES1 levels were increased in children with obesity diagnosed as MASLD and MetS. This study also suggested that serum levels of CES1 had an association with the risk of MASLD, MetS and could be used as a biomarker for predictive value.

## Electronic supplementary material

Below is the link to the electronic supplementary material.

Supplementary Material 1

## Data Availability

The data used to support the findings of this study are included within the article and its additional files.

## Abbreviations

MASLD: Metabolic dysfunction associated steatotic liver disease
MetS: Metabolic syndrome
WC: Waist circumference
WHtR: Waist to height ratio
SBP: Systolic blood pressure
DBP: Diastolic blood pressure
BMI: Body mass index
ALT: Alanine aminotransferase
AST: Aspartate aminotransferase
TG: Total triglyceride
TC: Total cholesterol
HDL-C: High-density lipoprotein cholesterol
LDL-C: Low-density lipoprotein cholesterol
FBG: Fasting blood glucose
FBI: Fasting blood insulin
HOMA-IR: Homeostasis model assessment of insulin resistance
IGF-1: Insulin-like growth factor 1
GDF15: Growth differentiation factor 15
CES1: Carboxylesterase 1
OR: Odds ratio
CI: Confidence interval
AUC: Area under the curve
