# Supplementary material for: Increased serum carboxylesterase-1 levels are associated with metabolic dysfunction associated steatotic liver disease and metabolic syndrome in children with obesity
Source: Ital J Pediatr. 2024 Sep 4;50:162. doi: 10.1186/s13052-024-01733-7 (PMC11373257; doi:10.1186/s13052-024-01733-7)
Supplement: Supplementary file 1 — Supplementary Material 1 [file 13052_2024_1733_MOESM1_ESM.doc]

**Extend Tabel 1. Association between serum carboxylesterase 1 levels and metabolic risks factors**

| **Variable** | **Unadjusted** | | **Adjusted [[1]](#footnote-2)** | |
| --- | --- | --- | --- | --- |
|  | **R** | ***P* value** | **R** | ***P* value** |
| Age | 0.265 | 0.024* | - | - |
| Weight [[2]](#footnote-3) | 0.259 | 0.028* | 0.119 | 0.325 |
| WC ^2^ | 0.262 | 0.026* | 0.191 | 0.113 |
| WHtR | 0.224 | 0.058 | 0.223 | 0.064 |
| BMI | 0.282 | 0.017* | 0.195 | 0.105 |
| BMI *Z*-score | 0.182 | 0.125 | 0.206 | 0.087 |
| ALT ^2^ | 0.468 | <0.001* | 0.517 | <0.001* |
| AST ^2^ | 0.431 | <0.001* | 0.480 | <0.001* |
| TG ^2^ | 0.458 | <0.001* | 0.446 | <0.001* |
| TC | 0.280 | 0.017* | 0.328 | 0.006* |
| HDL-C | -0.390 | <0.001* | -0.322 | 0.007* |
| LDL-C | 0.410 | <0.001* | 0.415 | <0.001* |
| FBG ^2^ | 0.118 | 0.324 | 0.065 | 0.592 |
| FBI ^2^ | 0.352 | 0.002* | 0.284 | 0.017* |
| HOMA-IR ^2^ | 0.381 | <0.001* | 0.305 | 0.010* |
| Adiponectin ^2^ | -0.355 | 0.002* | -0.294 | 0.013* |
| Leptin ^2^ | 0.282 | 0.016* | 0.214 | 0.075 |
| GDF15 ^2^ | 0.363 | 0.002* | 0.306 | 0.010* |
| IGF-1 | -0.315 | 0.007* | -0.458 | <0.001* |

1. Adjusted for age and sex. [↑](#footnote-ref-2)
2. Performed a logarithmic transformation.

   Notes: Pearson's correlations were performed to conform the correlation of CES1 with metabolic risk factors. ^*^*P* < 0.05. [↑](#footnote-ref-3)
